# The use of chatbots in university EFL settings: Research trends and pedagogical implications

**DOI:** 10.3389/fpsyg.2023.1131506

**Published:** 2023-03-22

**Authors:** Blanka Klímová, Prodhan Mahbub Ibna Seraj

**Affiliations:** ^1^Department of Applied Linguistics, Faculty of Informatics and Management, University of Hradec Kralove, Hradec Kralove, Czech Republic; ^2^Department of English, American International University-Bangladesh, Dhaka, Bangladesh

**Keywords:** chatbots, artificial intelligence, EFL, tertiary level, language skills, learning

## Abstract

This mini-review aims to identify major research trends, models, and theories and provide specific pedagogical implications for teaching when using chatbots in EFL classes. This study follows the guidelines of the PRISMA methodology and searches for open-access empirical studies in two reputable databases, Web of Science and Scopus. The results of this mini-review confirm the findings of other research studies, which show that the present research on the use of chatbots in university EFL settings focuses on their effectiveness, motivation, satisfaction, exposure, and assessment. The key contribution of this study lies in its evaluation of the chatbot’s potential in applying and integrating the existing theories and concepts used in EFL teaching and learning, such as CEFR, mind mapping, or self-regulatory learning theory. This will address the gap in the literature because no previous review study has conducted such an analysis. Overall, the findings of this mini-review contribute with their specific pedagogical implications and methods to the effective use of chatbots in the EFL environment, be it formal or informal.

## 1. Introduction

In the era of IoT (Internet of Things), different forms and shapes of AI (Artificial Intelligence) are being used to facilitate foreign language teaching and learning at different levels of education in different EFL contexts (Junaidi et al., 2020; Fitria, 2021). Chatbots represent such AIs employed at the macro and micro level in the class for developing learners’ different language skills, e.g., speaking, reading, listening, and writing (Gayed et al., 2022). Chatbots refer to a dialog system replicating written and/or verbal communication with human users, typically over the Internet. The dialogue system can be text-based or task-based and respond with speech, graphics, virtual gestures, or physically assisted tactual gestures (Belda-Medina and Calvo-Ferrer, 2022). In addition, thanks to their automated responses, they are becoming effective in language classes (Smutny and Schreiberova, 2020).

Language acquisition happens through interaction with peers, teachers, and other professionals (Çakıroğlu, 2018). Interaction is crucial for the language acquisition process because it gives learners comprehensible input, feedback on their output, and the chance to produce modified output (Liu, 2022). Such opportunities for language learning can be offered to learners through interaction with pedagogical or conversational chatbots (Yin and Satar, 2020; Mageira et al., 2022). AI chatbots have an impact on students’ communication abilities (Kim et al., 2021). Using text, speech, graphics, haptics, and gestures, as well as other modes of communication, chatbots assist students in completing educational tasks (Kuhail et al., 2022). The greatest strengths of chatbots are their usability and accessibility; their conversational metaphor and text-or voice-based interfaces make them more intuitive and mobile-friendly. Text-based interactions between humans and chatbots have demonstrated their potential benefits (Adam et al., 2021). Specifically, chatbots can instill in their users higher levels of motivation and engagement, which are crucial in technology-supported language learning (Petrović and Jovanović, 2021). The chatbots have allowed language learners to practice their language skills in real life. It is undoubtedly a useful tool for EFL students as they have few opportunities to use the target language in actual conversation. Moreover, for the sake of each participant’s unique language proficiency, an AI chatbot can help students learn by adapting how the lessons are delivered (Nghi et al., 2019). Overall, AI chatbots offer students a much wider range of services, improve learners’ motivation, and broaden the learners’ frame of conventional-based learning in the digital world, which is essential in the 21st century (Junaidi et al., 2020; Mukhallafi, 2020).

Existing literature review studies attempted to provide an overview of different aspects of implementing chatbots in education. For example, Kuhail et al. (2022) focused on the application of chatbot-learner interaction design techniques in education. On the other hand, Petrović and Jovanović (2021) or Huang et al. (2022) pointed out some approaches to using chatbots for language learning. Nevertheless, they mainly focused on their use in education in general without any specific recommendations for language practitioners. The same is true for the study by Ebadi and Amini (2022), whose authors concentrated on the role of social presence and human likeness on learner motivation when using a chatbot. Furthermore, another review study by Wollny et al. (2021) explored the pedagogical roles of chatbots, the use of chatbots for mentoring purposes, and their potential to personalize education. Thus, the existing literature review studies have not explicitly concentrated on the application of chatbots for learning English as a foreign language at the tertiary level. Furthermore, the existing literature reviews did not explore models and theoretical frameworks for using chatbots for English language learning and teaching. For implementing AI chatbots for teaching language skills in language classes, policymakers and teachers must come across models and frameworks for getting benefits. Therefore, this study aims to fill the gap in assisting language teachers, learners, and policymakers to use chatbots in their classes to develop EFL learners’ language skills and provide specific pedagogical implications for teaching when using chatbots in university EFL classes.

The following research questions were set in order to answer the aim of this study:

- *What were the publication trends and major research trends in the identified empirical studies on the application of chatbots for EFL learning and teaching at the tertiary level?*
- *What models and theoretical frameworks were used in these studies on the application of chatbots for EFL learning and teaching at the tertiary level?*

## 2. Methods

For the purpose of choosing more pertinent research studies on the topic and organizing them for a close examination, the Preferred Reporting Items for Systematic Reviews and Meta-Analyses (PRISMA) guidelines were followed (Moher et al., 2015; Ibna Seraj and Habil, 2021). Based on the guidelines of PRISMA, the search was not limited by a time scope but finished on 7 October 2022. In addition, only experimental studies dealing exclusively with the use of chatbots in teaching English as a foreign language with a special focus on their implementation in EFL classrooms were included in this mini-review. Theoretical, descriptive, observational, and non-experimental studies were excluded from the search as the main aim was to look for empirically verified findings. The search was conducted in two well-established databases, i.e., Scopus and Web of Science, in the titles of the articles, their abstracts, and keywords as this is sufficient to generate a reliable and adequate core of articles to be further analyzed.

### 2.1. Inclusion criteria

- Only experimental studies focusing on the research topic
- Published until October 2022
- Scopus and Web of Science databases
- Peer-reviewed and only English-written journal articles were included
- Search terms were applied in the title, abstract, or keywords of the articles
- Experimental studies with specific practical outcomes for ELL
- Open access

### 2.2. Exclusion criteria

- Descriptive studies, theoretical studies, conference proceedings, case studies, qualitative studies, observational studies
- Other than open-access studies
- Other (less reputable) databases
- Other languages
- The studies of the research scope, i.e., those which did not investigate the use of chatbots in EFL at a university level, e.g., Yang et al. (2022)

The gist of the exclusion criteria indicates that this study excluded all types of review studies, qualitative studies, and short studies like theoretical studies, conference proceedings, and case studies. It also did not consider non-open access and studies published in databases other than Scopus and Web of Science. Finally, studies published in languages other than English or not dealing with English language teaching and learning at the university level were excluded.

### 2.3. Search string

(“Chatbots OR “conversational agents” OR “virtual assistant”) AND (“English language” OR “foreign language”) AND “university.”

The initial search using this search string generated 110 documents from Scopus and 101 studies from the Web of Science. After applying all inclusion and exclusion criteria and removing duplicates, seven studies were fully analyzed since they covered all inclusion and exclusion criteria.

## 3. Results

The results of this mini-review in response to research questions (a) and (b) are discussed below.

Research question (a): *What were the publication trends and major research trends in the identified empirical studies on the application of chatbots for EFL learning and teaching at the tertiary level?*

### 3.1. Publications and research trends

#### 3.1.1. Publication trends

Table 1 presents the summary of the publication trends of empirical studies (*n* = 7) by researchers, contexts, methods, objectives, participants, instruments, and findings. In terms of researchers, we found that there were 15 researchers who conducted empirical studies on the application of chatbots in English language learning and teaching. This study reported that the empirical studies were conducted in 7 different contexts in Asian and European countries and Egypt.

**Table 1:** Summary of publication trends of the existing empirical studies.

Researchers	Contexts	Methods	Objectives	Participants	Instrument	Findings
Belda-Medina and Calvo-Ferrer (2022)	Spain and Poland	Mixed	The purpose of this study is to investigate how well students understand and feel Chatbots have contributed to their language education.	176 undergraduates	Pre-and post-tests, survey	Learners’ perceptions of Chatbot’s ease of use (PeU) and attitudes (AT) toward its use in language learning were both positive.
Hew et al. (2022)	China	Mixed	The objective of this study is to investigate the feasibility of utilizing chatbots to facilitate student-driven goal setting and online community building.	29 postgraduate students and 38 undergraduate students	Survey, open-ended interview	In terms of perceived utility and usability, learners had positive experiences with chatbots.
Kim et al. (2021)	Korea	Quantitative	The goal of this study is to determine whether or not the use of artificially intelligent chatbots improves students’ confidence and enthusiasm in speaking skills.	49 university students	Pre-and post-tests, survey	The speaking skills of learners are greatly enhanced by the use of chatbots.
Lin and Mubarok (2021)	Taiwan	Quantitative	The goal of using AI chatbots in an English-speaking classroom is to improve students’ oral communication and classroom participation.	50 students	Pre-and post-test, survey	In comparison to the traditional AI chatbot approach, the MM-AI AI chatbot approach more effectively promoted the student speaking performances (C-AI).
Mahmoud (2022)	Egypt	Quantitative	To investigate the effect of AI-based conversational chatbots on the engagement and improvement of learners’ speaking skills.	156 undergraduate students	Pre-and post-test, survey	The integration of conversational chatbots has a positive effect on the speaking performance of EFL learners.
Nghi et al. (2019)	Vietnam	Quantitative	Using chatbots to help students learn a particular skill in a foreign language by connecting their interests, activities, and performances.	200 Undergraduate students	Placement test	The majority of students believed that using AI chatbots as an essential part of their learning process to create excitement and fun.
Yin and Satar (2020)	China	Quantitative	Examining Mitsuku, a conversational chatbot, and Tutor Mike, a pedagogical chatbot, for their usefulness in learning a foreign language.	8 Chinese learner	Questionnaire	The results of this study suggest that while students with lower language skills would gain the most from interacting with pedagogical agents, students with higher skills were less satisfied with chatbots and showed less engagement with the pedagogical agent.

The majority of studies employed quantitative research design (*n* = 5) using survey questions and tests and reported findings from descriptive statistical analysis. The objective of the studies was to investigate the impact of chatbots on learners’ language skills development. These empirical studies employed 668 university learners to elicit the effect of chatbots on English language learning. The average number of participants in each study was 96. Finally, the findings indicate that the application of chatbots in EFL teaching and learning positively affected the development of learners’ language skills.

#### 3.1.2. Major research trends of the studies

**Figure 1:**
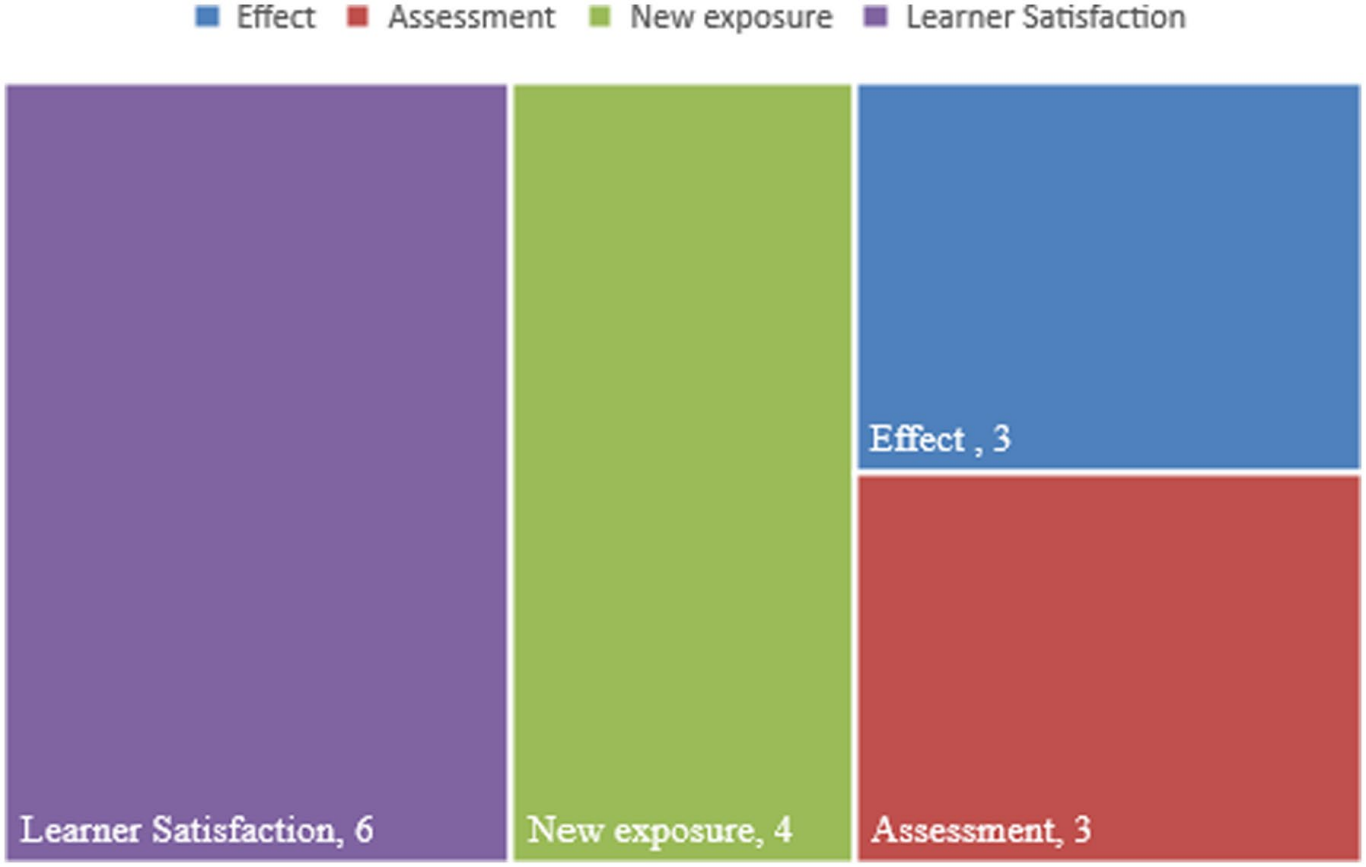
Major research trends.

The major research trends are presented in Figure 1 and discussed in detail in the sections below.

Our review shows that there are several different research trends using AI chatbots in the classroom for language teaching and learning, e.g., learner satisfaction, effects, new exposure for learning, and assessment of language performance.

##### 3.1.2.1. Effects of the application of AI Chatbots

We found that the application of AI chatbots has several effects on learners’ performance in learning different language skills. Kim et al. (2021) showed that learners improved in terms of using intonation, stress, and fluency in speaking skills after using chatbots. The findings revealed that chatbots enhanced students’ engagement in developing speaking skills inside and outside the classroom (Mahmoud, 2022). Moreover, for learning specific language skills, a chatbot generates excitement and fun while learning (Nghi et al., 2019). In addition, the mind map-guided AI chatbot approach (MM-AI) promoted student English-speaking performances through interaction between the robots and humans (Lin and Mubarok, 2021). Nonetheless, there were both negative and positive effects according to the learners’ level. The students with low-level language skills benefited the most, whereas the learners with high-level language skills felt dissatisfied using it (Yin and Satar, 2020). Overall, the findings from the detected experimental studies indicated that there had been a significant positive effect of using chatbots on learners’ learning of language skills.

##### 3.1.2.2. New exposure

We found that AI chatbots that AI chatbots have recently been used in the classroom. Thus, the chatbot is a platform for both teachers’ and learners’ new exposure to teaching and learning different language skills in different contexts (Adam et al., 2021). This is especially true for developing EFL speaking skills (Divekar et al., 2021). This conversational agent works by providing visual contexts, embodying dialogue agents, speech recognition, sustained engagement, and understanding natural language for improving conversational skills (Adam et al., 2021; Allouch et al., 2021; Fitria, 2021).

##### 3.1.2.3. Learner satisfaction

Another vital theme derived from the analysis was the satisfaction of using chatbots for teaching and learning different language skills. Belda-Medina and Calvo-Ferrer (2022) investigated participants’ level of satisfaction employing the Chatbot–Human Interaction Satisfaction Model (CHISM) and found positive perception of the integration of conversational agents in language learning in terms of participants’ perceived ease of use (PeU) and attitudes (AT). In support of satisfaction, Hew et al. (2022) found positive learner experiences concerning the chatbots’ perceived usefulness and ease of use. This was also true for the study by Mahmoud (2022).

##### 3.1.2.4. Assessment

This study also found that AI Chatbots are used to assess learners’ language skills. Salamanca (2019) also reported that a chatbot capable of assessing the user’s level of proficiency in the CEFR framework while having a natural conversation is held. The application of chatbots assists in automated assessment, short answers assessment, and language assessment of learners’ language performance, which has been confirmed by other review studies on the use of chatbots in education (Pérez et al., 2020; Wollny et al., 2021; Huang et al., 2022). Thus, it is reported that the use of chatbots for the assessment of learners’ performance is effective.

Research question (b): *What models and theoretical framework were employed in the detected studies on the application of chatbots for EFL learning and teaching at the tertiary level?*

### 3.2. Integration with models and theories

We found that there were different conceptual models and theories proposed and implemented for using chatbots to develop learners’ language development. Most of the studies employed models and theories to facilitate teaching and learning, and only CEFR was employed to assess learners’ language performance. These are presented as follows:

#### 3.2.1. Common European framework of reference

For automated assessment, short answers assessment, and language assessment, the application of a chatbot in the framework of the common European framework of reference (CEFR) works effectively (Salamanca, 2019). For automated assessment, this approach defines a set of quantifiable characteristics, such as a word count or essay length, and employs multiple linear regressions to forecast the essay score. For short answer assessment, lexical similarity evaluates how closely two words or phrases resemble one another, and semantic similarity extracts data about the semantic separation between words from a data set (typically WordNet). Therefore, a chatbot can assess the user’s level of language proficiency within the CEFR framework while conversing naturally with them (Pérez et al., 2020; Wollny et al., 2021; Huang et al., 2022).

#### 3.2.2. Mind mapping strategy

We discovered that mind mapping strategy using AI chatbots provides EFL learners with a practicing environment inside and outside the classroom by identifying the meanings of users’ statements and responding accordingly (Lin and Mubarok, 2021). This strategy processes words into a picture with a core word at the center or the top and related words or images linked with the keywords by lines. Students who use mind mapping perform better academically and can think more logically (Swestyani et al., 2018). In addition to improving students’ academic performance and organizing interactions between humans and robots, the MM-AI Chatbot (Mind Mapping) is more effective than the C-AI (Conventional AI) chatbots (Lin and Mubarok, 2021; Mageira et al., 2022).

#### 3.2.3. Self-regulatory learning

In this mini-review, we pointed out that self-regulatory learning was facilitated through the application of AI chatbots in language classrooms. Using adaptive learning environments and intelligent tutoring systems, chatbots encourage self-regulated learning by enhancing the individual learner’s experience (Mahmoud, 2022). The dynamic process of self-regulated learning, which is comprised of cognitive, affective, motivational, and behavioral components, allows learners to control their own learning (Panadero, 2017).

#### 3.2.4. Situated learning theory

We showed that chatbots facilitated situated learning providing authentic settings and contexts, whether inside or outside the classroom (Mahmoud, 2022). Here, learners gain the necessary knowledge by taking advantage of every opportunity in a natural and realistic setting. As Chatbots are conversational agents, learners interact with others primarily through language, and conversation creates learning in the class and out of the class (Adamopoulou and Moussiades, 2020; Belda-Medina and Calvo-Ferrer, 2022). The advantage of chatbots in education is improving the learning experience (Okonkwo and Ade-Ibijola, 2021).

#### 3.2.5. SMART framework

We reported that using the SMART framework for implementing AI chatbots for learning and teaching language skills was effective. Supporting student goal-setting and social presence to develop listening skills, the chatbots were useful through the SMART (specific, measurable, achievable, realistic, and timely) goal-setting framework (Hew et al., 2022). Both the learning buddy chatbot and the goal setting Chatbot employing Google Dialogflow were visual development tools that did not require prior computer programming knowledge (cf. Mendoza et al., 2022).

#### 3.2.6. Socratic IM

The chatbots facilitated the Socratic Inquiry method in EFL group discussions, increasing learners’ critical thinking, satisfaction, and number of conversations (Belda-Medina and Calvo-Ferrer, 2022). Furthermore, the chatbot online group discussion enhanced learners’ collaborative learning activity and cognitive engagement in discussions (Hew et al., 2022; Kuhail et al., 2022). We also showed that the students communicated more effectively by using English to communicate in the form of Socratic IM with the Chatbot (Belda-Medina and Calvo-Ferrer, 2022).

## 4. Discussion

Our mini-review show that only 7 open access empirical studies were conducted at the tertiary level (Nghi et al., 2019; Yin and Satar, 2020; Kim et al., 2021; Lin and Mubarok, 2021; Belda-Medina and Calvo-Ferrer, 2022; Hew et al., 2022; Mahmoud, 2022). The oldest one dates back as late as 2019, which indicates that the use of chatbots in university EFL classrooms is quite novel, and the number of these studies reveal that the research on their applications is scarce (Yin and Satar, 2020; Belda-Medina and Calvo-Ferrer, 2022; Merelo et al., 2022). Nevertheless, all the studies found that chatbots had a positive impact on the development of learners’ English language skills, especially on developing learners’ speaking skills in terms of suprasegmental features, e.g., intonation, and stress, as well as fluency engaging learners in practice inside and outside the classes (Kim et al., 2021; Mahmoud, 2022; or Lin and Mubarok, 2021), as well as facilitating peer and group activities among learners (Mahmoud, 2022). However, we indicated that more research should be done among low-level foreign language learners since these benefit from using chatbots the least (Yin and Satar, 2020) to address the gaps in the literature.

Moreover, we confirm the findings of other research studies (e.g., Adamopoulou and Moussiades, 2020; Chu et al., 2022; or Mageira et al., 2022), which show that the use of chatbots in an educational setting is effective, motivating, satisfying for EFL learners because it provides more exposure to the target language as they are accessible anywhere and anytime (Chen et al., 2020). Furthermore, they offer situated context, as well as immediate automated feedback that can reduce teachers´ load.

Our key contribution of this study, however, lies in its evaluation of chatbot potential in applying and integrating the existing theories and concepts used in EFL teaching and learning, such as CEFR, mind mapping, or self-regulatory learning theory (e.g., Ebadi and Amini, 2022; Huang et al., 2022; Kuhail et al., 2022; or Wollny et al., 2021). Thus, the findings of this study may help EFL teachers develop students’ language skills and structures using the AI Chatbots application. In addition, the results of this study should be of interest to all stakeholders, e.g., EFL teachers, chatbot developers, the academic community, or policymakers, i.e., those involved in any aspect of their implementation in EFL classrooms.

We set several pedagogical implications for EFL practitioners, which are as follows:

- chatbots are ideal for informal settings since they provide authentic context and can be used anytime and anywhere
- chatbots are user-friendly; therefore, easy to use (no technical knowledge is required) and engaging
- short conversations are more suitable when providing automated assessment
- in particular, speaking skills can be developed, and students with a low level of foreign language benefit the most
- chatbots should be used since they enhance the critical thinking of EFL learners through Socratic IM
- AI chatbots play a significant role in reducing language-learning inhibitions and speech-related fears in English as a foreign language (cf. Bao, 2019)

The major limitation of this study seems to be a lack of empirical research on this topic. Nevertheless, it provides valuable, up-to-date information for further empirical and theoretical research in this area. Undoubtedly, the findings of this mini-review contribute with their practical implications and methods to the effective use of chatbots in the EFL environment, be it formal or informal.

## Author contributions

All authors listed have made a substantial, direct, and intellectual contribution to the work and approved it for publication.

## Funding

This study was supported by the Excellence project 2023, run at the Faculty of Informatics and Management of the University of Hradec Kralove, Czech Republic.

## Conflict of interest

The authors declare that the research was conducted in the absence of any commercial or financial relationships that could be construed as a potential conflict of interest.

## Publisher’s note

All claims expressed in this article are solely those of the authors and do not necessarily represent those of their affiliated organizations, or those of the publisher, the editors and the reviewers. Any product that may be evaluated in this article, or claim that may be made by its manufacturer, is not guaranteed or endorsed by the publisher.
